# The rheumatoid arthritis treat-to-target trial: a cluster randomized trial within the Corrona rheumatology network

**DOI:** 10.1186/1471-2474-15-389

**Published:** 2014-11-21

**Authors:** Leslie R Harrold, George W Reed, J Timothy Harrington, Christine J Barr, Katherine C Saunders, Allan Gibofsky, Jeffrey D Greenberg, Ani John, Jenny Devenport, Joel M Kremer

**Affiliations:** Department of Orthopedics, University of Massachusetts Medical School, Worcester, MA 01655 USA; University of Massachusetts Medical School, Madison, WI USA; Corrona, LLC, Southborough, MA USA; Hospital for Special Surgery, New York, NY USA; NYU Hospital for Joint Diseases, New York, NY USA; Genentech, Inc, South San Francisco, CA USA; Albany Medical College and the Center for Rheumatology, Albany, NY USA

**Keywords:** Treat to target, Rheumatoid arthritis, Corrona, Usual care

## Abstract

**Background:**

The treat-to-target (T2T) approach to the care of patients with rheumatoid arthritis involves using validated metrics to measure disease activity, frequent follow-up visits for patients with moderate to high disease activity, and escalation of therapy when patients have inadequate therapeutic response as assessed by standard disease activity scores. The study described is a newly launched cluster-randomized behavioral intervention to assess the feasibility and effectiveness of the T2T approach in US rheumatology practices. It is designed to identify patient and provider barriers to implementing T2T management. This initial paper focuses on the novel study design and methods created to provide these insights.

**Methods/Design:**

This trial cluster-randomizes rheumatology practices from the existing Corrona network of private and academic sites rather than patients within sites or individual investigators to provide either T2T or usual care (UC) for qualified patients who meet the 2010 revised American College of Rheumatology criteria for the diagnosis of rheumatoid arthritis and have moderate to high disease activity. Specific medication choices are left to the investigator and patient, rather than being specified in the protocol. Enrollment is expected to be completed by the end of 2013, with 30 practices randomized and enrolling a minimum of 530 patients. During the 12-month follow-up, visits are mandated as frequently as monthly in patients with active disease in the T2T group and every 3 months for the UC group. Safety data are collected at each visit. The coprimary endpoints include a comparison of the proportion of patients achieving low disease activity in the T2T and UC groups and assessment of the feasibility of implementing T2T in rheumatology practices, specifically assessment of the rates of treatment acceleration, frequency of visits, time to next visit conditional on disease activity, and probability of acceleration conditional on disease activity in the 2 groups.

**Discussion:**

This cluster-randomized behavioral intervention study will provide valuable insights on the outcomes and feasibility of employing a T2T treatment approach in clinical practice in the United States.

**Trial registration:**

NCT01407419

**Electronic supplementary material:**

The online version of this article (doi:10.1186/1471-2474-15-389) contains supplementary material, which is available to authorized users.

## Background

An estimated 1.3 million people in the United States are affected by rheumatoid arthritis (RA), a chronic, progressive, inflammatory disease that causes pain, joint damage, and disability [1]. It is estimated that 20% to 30% of patients with early RA become permanently disabled during the first 2 to 3 years of the disease [2]. The ultimate goal of treatment is to achieve remission of active inflammation, given that a substantial proportion of patients show radiographic progression even while in a state of low disease activity (LDA) [3, 4]. Remission is now possible in many patients with RA treated early and aggressively with an increasing array of effective medications. Treatment generally starts with traditional nonbiologic disease-modifying antirheumatic drugs (DMARDs), and if remission is not achieved, nonbiologic DMARDs can be combined and/or biologic DMARDs can be initiated [5].

There is a growing consensus that rheumatologists should measure patient disease activity using validated composite clinical measures and then accelerate treatment until the disease is in remission, an approach referred to as treat-to-target (T2T) management [6]. Studies comparing T2T management with usual care (UC) have clearly shown T2T to be superior. The Tight Control of Rheumatoid Arthritis (TICORA) trial demonstrated an advantage of T2T care using only standard DMARDs with mandatory acceleration every 3 months in selected patients with early RA [7]. Findings from the TICORA trial have since been confirmed in multiple other studies of tight control treatment strategies, including data from the Dutch Rheumatoid Arthritis Monitoring registry and the Computer Assisted Management in Early Rheumatoid Arthritis (CAMERA) trial [8, 9]. These trials were performed in Europe, where access to biologic agents is significantly restricted compared with the United States [10].

The feasibility and advantages of T2T management relative to UC still need to be verified in US rheumatology practices, in more representative US RA populations, and in the absence of specific protocols that rigidly define treatment choices, which may not be practical in routine care. For these reasons, a US-based, independent observational registry (Corrona, LLC) has designed and launched a behavioral intervention clinical trial, cluster-randomized by practice sites into T2T and UC management, for patients with moderate to high RA disease activity at enrollment. This initial paper focuses on the novel study design and methods created to evaluate these research questions and the rationale underlying these methodological decisions. The feasibility assessments of visits and treatment accelerations mandated by this protocol, and the barriers to implementing the treatment protocols, are critical to understanding whether practicing US rheumatologists will be able to successfully and routinely apply T2T management.

The Corrona T2T trial is enrolling patients with moderate to high disease activity as measured by the Clinical Disease Activity Index (CDAI), an instrument commonly used in the US and recommended by the American College of Rheumatology for evaluation of disease activity [11]. The trial mandates assessment of disease activity with monthly visits in the T2T intervention group and up to monthly treatment acceleration until disease control is achieved. The Corrona study does not recommend any specific medication treatments—rather a variety of traditional and biologic DMARD options may be employed at the discretion of the treating rheumatologist investigator (Table 1). The UC sites are required only to provide a Corrona RA report every 3 months for visits that took place for their enrolled patients, and treatments and visit schedule for a patient are determined at the discretion of the individual rheumatologist.

**Table 1:** **Comparison of features of TICORA, BeSt, and Corrona T2T with EULAR recommendations**[12]

	TICORA	BeSt	Corrona T2T	EULAR
Mandated acceleration	Yes	Yes	Yes	Yes
Biologics accessible	No	Yes	Yes	Yes
Predefined acceleration order of therapeutic options	Yes	Yes	No	No
Biologic prevalence in RA population ≥20%	No	No	Yes	No
Unique cost-effectiveness climate	Yes	Yes	Yes	Yes

BeSt: Behandel Strategieen (study); EULAR, European League Against Rheumatism; RA, rheumatoid arthritis; T2T, treat to target; TICORA: Tight Control of Rheumatoid Arthritis.

Corrona, LLC was founded in 2001 and currently includes ≥160 rheumatology practices (80% private practice) and ≥600 rheumatologists across 40 states in the United States. The registry collects longitudinal “real-world” data from patients and their treating rheumatology providers during routine clinical visits. As of March 3rd, 2014, data have been collected on more than 39,956 patients with rheumatologist-diagnosed RA. Patient- and provider-reported data collected at each Corrona registry visit include RA disease severity and activity; present and past RA treatments with doses, comorbidities, adverse events, and selected laboratory and imaging results; sociodemographic information; and a range of patient-reported outcomes.

## Methods/Design

### Objective and design

**Figure 1:**
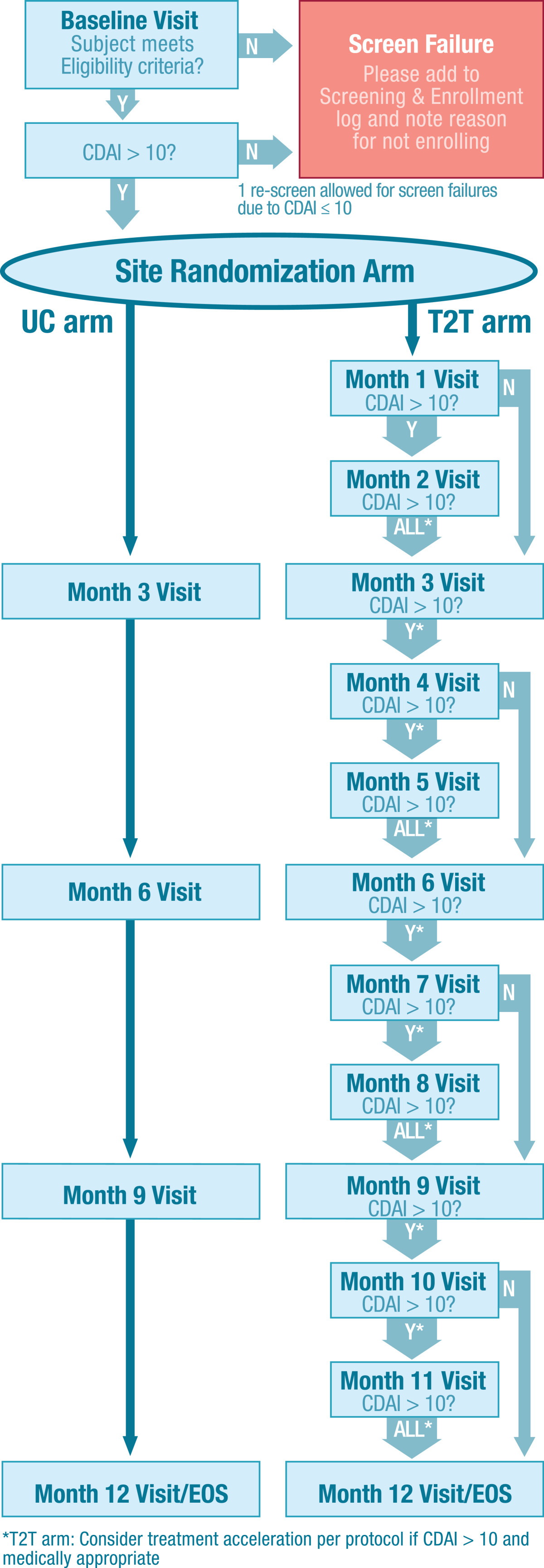
**The flow diagram of the treat-to-target study.** CDAI, Clinical Disease Activity Index; EOS, end of study; T2T, treat to target; UC, usual care.

The T2T study is a 12-month trial with cluster randomization. The protocol mandates increased visit frequency and treatment acceleration in patients with active disease at T2T sites but not at UC sites, where visits and collection of routine Corrona data are required every 3 months (Figure 1). Cluster-randomized trials are characterized by randomization at the level of the cluster or group—in the case of this study, the cluster was clinic sites [13–15]. All individuals in the cluster were assigned to the same study arm. The approach was used because it would be difficult, if not impossible, for a doctor to treat patients differently based on treatment assignment—i.e., treating some patients to target while giving “usual care” to others.

The coprimary objectives of the study are to (1) examine achievement of LDA in each site’s enrolled population based on the CDAI at 12 months (CDAI ≤10) and (2) assess the feasibility of a T2T approach by comparing rates of treatment acceleration, visit frequency conditional on disease activity, and probability of treatment acceleration conditional on disease activity in the T2T group compared with the UC group at 12 months. Secondary objectives are to (1) assess mediators of differences in LDA rates between groups, including specific medication uses and frequency of medication acceleration; (2) examine whether the impact of physician acceleration of medication use in those patients with active disease in both the T2T and UC groups is associated with achieving LDA; (3) determine the proportion of patients who achieve LDA at 6 months; (4) compare the performance of the 28-joint disease activity score via erythrocyte sedimentation rate (DAS28-ESR) and Routine Assessment of Patient Index Data 3 (RAPID3) at 6 and 12 months; (5) determine the frequency of ineligibility for treatment acceleration; (6) determine the rates of drug toxicities over 12 months; and (7) compare the rates of adverse events of special interest, termed *targeted adverse events*.

### Study site recruitment

For this study, we approached rheumatology practices that currently participate in Corrona as well as other practices not previously affiliated with Corrona. Sites already participating in the Corrona Effectiveness Registry to Study Therapies for Arthritis and Inflammatory Conditions (CERTAIN) comparative effectiveness trial were excluded so as not to interfere with the primary endpoint analyses in individual studies as the recruitment criteria in these studies are quite similar [16]. Sites that expressed interest in the trial were invited to review the protocol and complete a feasibility assessment after signing a nondisclosure agreement. Feasibility data were reviewed by the project team, and sites were formally invited to participate if they had the interest and capacity to meet the expectations for the trial. Some of the invited sites declined to participate in the study, and the reasons provided were tracked and included lack of adequate staff resources, current project load, institutional review board (IRB) concerns (primarily at academic sites), and other priorities of the sites (for example, transitioning to electronic medical records).

Sites were stratified by size and were asked to assess the number of participants they would enroll. Using a cutoff of 34 based on sample size and power calculations, the sites were classified as *large* or *small* for the purpose of randomization. Sites were then randomized in a 1:1 ratio to either the T2T or UC cluster after obtaining IRB approval. Approvals for the study were obtained from local institutional review boards of participating academic sites and a central institutional review board (New England IRB) for private practice sites. Site allocation remained sealed until after the participating physicians obtained IRB approval. Then sites received training materials and initiation training specific to their assignment. Patient recruitment began in July 2011.

### Physician and staff training

Investigators and staff at all participating sites received training by phone on the T2T trial protocol. The manual of procedures was reviewed with the principal investigator and site staff during this 30- to 60-minute training call, and questions about the protocol and trial activities were answered. T2T and UC sites received separate instructions specific to their visit and treatment acceleration expectations. In addition, training was supplemented as needed via e-mail, newsletter reminders, site feedback, and telephone discussions. Retraining was completed on an as-needed basis for sites that experienced ongoing challenges in adhering to the protocol and/or completing required clinical questionnaires. In addition to trial-specific training, investigators and site staff were documented to be up-to-date on Good Clinical Practices (GCP) training for research involving human subjects.

Protocol-defined inclusion and exclusion criteria were carefully reviewed with the physician investigators in both arms during the site-training teleconference before site activation to ensure that the target population was clearly defined. Investigators were also encouraged to call with any eligibility questions during the course of the trial. Misconceptions were addressed as detected, and feedback regarding challenges was regularly encouraged. Specific recruitment activities and levels at individual sites may vary based on available resources, provider schedules, workloads, and other considerations. Eligibility training and support was consistent across treatment arms.

### Patient recruitment

Patients with RA and active disease (CDAI >10) are invited to participate by their treating rheumatologists. Specific inclusion criteria are outpatients aged ≥18 years with RA according to the America College of Rheumatology (ACR) criteria, moderate to high disease activity based on the CDAI, and patient agreement with the schedule of study visits and provision of informed consent. Additionally, patients need to be deemed clinically and medically appropriate for treatment acceleration by the rheumatologist-investigator and willing to have their therapy escalated as appropriate. Patients were not excluded based on RA disease duration or prior medication use to ensure a real-world sample of participants.

Exclusion criteria are current or planned pregnancy and/or breastfeeding during the study period; planned surgery; ACR functional class IV; prednisone daily doses >10 mg within the prior 4 weeks; history of serious infection (defined as follows: (1) 1 hospitalization or use of parenteral antibiotics within the past 6 months, (2) ≥2 hospitalizations or courses of parenteral antibiotics within the past 12 months, (3) tuberculosis infection, (4) infection with human immunodeficiency virus, (5) hepatitis B virus infection, or (6) hepatitis C virus infection); serious comorbidities; chronic pain syndrome that either confounds or makes difficult the assessment of RA disease activity; and recent dose changes of traditional and biologic DMARDs that, in the opinion of the treating rheumatologist, have not had sufficient time to meaningfully impact disease activity.

### T2T intervention and UC sites

Patients enrolled in the T2T arm have mandated visits and treatment acceleration dependent on patient disease activity and physician discretion. As shown in Table 2, monthly visits are mandated as long as the patient continues to have a CDAI >10 (moderate to high disease activity), and treatment acceleration is mandated at least every 3 months until low disease activity is achieved or until end of the study (can be as frequently as monthly). Treatment acceleration is defined as a dosage increase in nonbiologic or biologic DMARDs, the addition or switching of nonbiologic or biologic DMARDs, or changing from oral to subcutaneous methotrexate administration. For infliximab, this includes a decrease in the dosing interval. In accordance with GCP, patients and investigators at the T2T sites may decide not to accelerate treatment, even if the CDAI criteria for acceleration are met, based on clinical reasons and/or patient preferences, as noted previously. Reasons for not accelerating treatment are recorded by the investigators. These include new or worsening comorbid conditions, anticipated lag in response time for recently initiated therapy, upcoming surgeries, pregnancy and/or breastfeeding, elevated CDAI deemed by the treating rheumatologist to be unrelated to RA (eg, a chronic or acute pain condition), and patient refusal.

**Table 2:** **Schedule of visits and laboratory assessments over the study period**

Procedure	Baseline	Monthly visits	Month 3	Month 6	Month 9	Month 12/End of study
Informed consent for T2T study	T2T, UC					
Corrona patient and physician enrollment questionnaires	T2T, UC					
Corrona patient and physician follow-up questionnaires		T2T	T2T, UC	T2T, UC	T2T, UC	T2T, UC
Baseline case report form	T2T, UC					
Study follow-up visit case report form		T2T	T2T, UC	T2T, UC	T2T, UC	T2T, UC
T2T-arm–only questionnaire	T2T	T2T	T2T	T2T	T2T	T2T
Corrona participant exit form (specify T2T study)						T2T, UC
Laboratory assessments						
Erythrocyte sedimentation rate	T2T, UC		T2T, UC	T2T, UC	T2T, UC	T2T, UC

T2T, treat to target; UC, usual care.

Patients in the UC arm are required to return for visits and complete Corrona reports every 3 months, but care is otherwise left to the discretion of the investigators at sites randomized to this arm. Patients at most sites are paid a nominal amount for their participation to compensate for the inconvenience of frequent visits and cover the cost of gas, and these payments were approved by the appropriate independent review boards.

### Clinical assessment

At each visit, 3 composite disease activity scores are collected, including CDAI, DAS28, and RAPID3. Erythrocyte sedimentation rate is determined locally every 3 months using the Westergren method. Frequencies of visits to the site by patients in both groups will be measured. All changes in nonsteroidal anti-inflammatory drugs, glucocorticoids, nonbiologic DMARDs, and biologics will be tracked throughout the course of the study. Additionally, any serious adverse events will be reported to Corrona within 48 hours, and the participating providers will be asked to report these as necessary to the US Food and Drug Administration and/or drug manufacturer. Targeted adverse events include anaphylaxis, cancers/malignancies, cardiovascular events (revascularization procedure, ventricular arrhythmia, cardiac arrest, myocardial infarction, acute coronary syndrome, unstable angina, congestive heart failure, stroke, and transient ischemic attack), gastrointestinal perforation, hospitalizations or biopsies for hepatic dysfunction, serious infection (infections requiring hospitalizations or intravenous antibiotics), neurological events (hospitalization, progressive multifocal leukoencephalopathy and other demyelinating diseases), and serious spontaneous bleeding requiring hospitalizations.

### Analysis plans

Coprimary endpoints will assess the differences between the randomized groups in physician behavior and patient disease activity. Specifically, we will compare the rates of physician visits (visits per patient-years of follow-up) and rates of acceleration (accelerations per visits with CDAI >10) between the T2T and UC sites to see if there are differences in the care approaches between the two treatment arms (e.g., visit frequency and tight control). In addition, we will compare achievement of LDA at 12 months from baseline by an intent-to-treat analysis. The unit of analysis for visit rates and LDA rates will be patient-clustered within physician and within site (the unit of randomization). Patients as well as physician treatment patterns will differ by site, but these differences should be balanced by site randomization. If large differences do exist in patient characteristics by arm even after site randomization, adjustment for those characteristics can be carried out. Random-effects regression models will be used to assess and account for correlations due to clustering. Assessment of differences in patient characteristics across sites will be made because the randomization was by site not by patient, and the comparison between randomized arms will be adjusted for differences. For analysis of acceleration, the unit of analysis will be a patient visit (with CDAI >10), which adds the additional clustering of visit within patient. Several secondary and exploratory analyses are planned. These include using the above mentioned methodology to evaluate the differences in the two treatment arms using the DAS28-ESR and the patient-reported outcomes based on the RAPID3 [17]. In addition, we will perform analyses that will focus on time to next visit when a patient has moderate to severe disease activity, rates of targeted adverse events, and differences in continual measures of disease activity. Specifically, rates of drug toxicities over 12 months will be calculated based on the reported number of toxicities per person-time of follow up using a random-effects Poisson regression model adjusting for the levels of clustering and potential confounders. We will estimate and test differences in continual measures of disease activity using random-effects linear regression models adjusting for the levels of clustering and compare mean changes in scores between the T2T and UC groups.

### Determination of sample size

A recruitment goal of 16 patients per site was determined to provide maximum analytic power for analysis of outcomes. Specifically, the choice of 15 sites per group with 16 patients per site was based on power estimates using intraclass correlation (ICC) estimates and estimated LDA rates observed in the Corrona registry, the possibility of dropped sites, and some imbalance in patients per site. The goal of recruiting 530 patients (265 per randomized arm) assumed a 10% dropout rate during the course of the study. With ICC =0.09 and the alternative hypothesis of 40% LDA vs 60% LDA at 1-year follow-up in the 2 randomized groups (control vs T2T), there is 80% power for 14 sites per group and 16 patients per site or 15 sites per group and 14 patients per site. Choosing 15 sites per group and 16 patients per site allowed for the possibility of loss of a site per group and any minor loss of power due to imbalance in patients per site—1% to 3%, with moderate imbalance [18].

## Discussion

The T2T clinical trial is a newly launched cluster-randomized behavioral intervention study examining the outcomes and feasibility of a T2T treatment paradigm in clinical practice in the United States. This includes evaluating whether patients and providers are able to adhere to a T2T protocol in terms of both visit frequency and treatment acceleration in those with persistent active disease. The established infrastructure of Corrona is used for patient enrollment, physician participation, and collection and storage of data. The commitment of Corrona investigators and enrolled patients to providing standardized, validated reports during routine visits is leveraged to perform this T2T clinical trial in which visits and laboratory evaluations are instead mandated at regular but different intervals at the T2T and UC sites.

Protocol development for this trial was interesting in several respects. Randomizing T2T and UC care by patients or individual investigators within the same site was rejected due to the likelihood of treatment protocol crossover. That is, it would be very challenging for the investigators at a site to treat the patients differently. Randomizing participating sites was seen as critical to addressing this issue and attempting to control for potential differences in disease activity measurement and treatment acceleration from site to site and potentially across investigators within sites. Given this novel approach, the Corrona statistical group was compelled to carefully consider optimal powering and analytic approaches. Using a cluster randomization trial design, the Corrona statistical group followed the methodologies described by Donner and Klar for power and sample size estimates and the use of mixed-effects models for analytic procedures [14]. The practical issues of both patient and site dropout and patient sample imbalance across sites were also addressed.

The major limitation of this trial may reside in the investigators’ discretion regarding treatment acceleration and treatments selected. UC investigators may elect to accelerate care and prescribe biologic DMARDs frequently, and T2T investigators may choose not to do so. In either case, the differences in treatments and disease activity measurements may be less than expected from historical Corrona registry data.

In summary, the T2T study harnesses the expertise and experience of Corrona’s scientific team and existing network to evaluate whether T2T improves outcomes without compromising safety and how to incorporate T2T treatment into clinical practice. We look forward to communicating the results of this novel trial.

## Authors’ original submitted files for images

Below are the links to the authors’ original submitted files for images. Authors’ original file for figure 1

## Abbreviations

ACR: American College of Rheumatology
BeSt: Behandel Strategieen (study)
CDAI: Clinical Disease Activity Index
DAS28: Disease activity score of 28 joints
DMARD: Disease-modifying antirheumatic drug
ESR: Erythrocyte sedimentation rate
EULAR: European league Against Rheumatism
GCP: Good Clinical Practice
ICC: Intraclass correlation
IRB: Institutional review board
LDA: Low disease activity
RA: Rheumatoid arthritis
RAPID3: Routine Assessment of Patient Index Data 3
T2T: Treat to target
TICORA: Tight Control of Rheumatoid Arthritis
UC: Usual care.
