# Supplementary figures and images for: The rheumatoid arthritis treat-to-target trial: a cluster randomized trial within the Corrona rheumatology network
Source: BMC Musculoskelet Disord. 2014 Nov 21;15:389. doi: 10.1186/1471-2474-15-389 (PMC4258022; doi:10.1186/1471-2474-15-389)

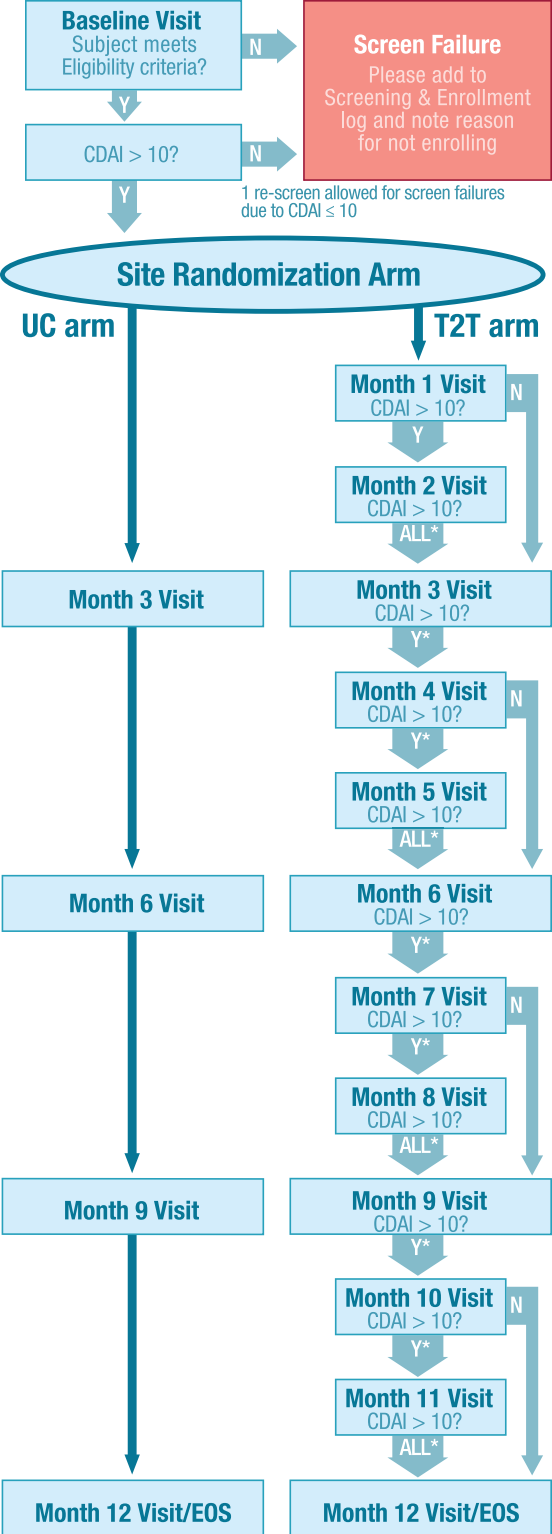

\*T2T arm: Consider treatment acceleration per protocol if CDAI  $> 10$  and medically appropriate

Supplement: Supplementary file 1 — Authors’ original file for figure 1 [file 12891_2013_2337_MOESM1_ESM.pdf]
